# Cost of TB services in healthcare facilities in Kenya

**DOI:** 10.5588/ijtld.21.0129

**Published:** 2021-12-01

**Authors:** A. Kairu, S. Orangi, R. Oyando, E. Kabia, P. Nguhiu, J. Ong'ang'o, N. Mwirigi, Y. V. Laurence, N. Kitson, I. Garcia Baena, A. Vassall, E. Barasa, S. Sweeney, L. Cunnama

**Affiliations:** 1 Health Economics Research Unit, KEMRI-Wellcome Trust Research Programme, Nairobi, Kenya; 2 Centre for Respiratory Diseases Research, Kenya Medical Research Institute, Nairobi, Kenya; 3 Ministry of Health, Division of National Tuberculosis, Leprosy, and Lung Disease Program, Nairobi, Kenya; 4 Department of Global Health and Development, Faculty of Public Health and Policy, Centre for Health Economics in London, London School of Hygiene & Tropical Medicine, London, UK; 5 Nuffield Department of Medicine, University of Oxford, Oxford, UK; 6 TB Monitoring and Evaluation, Global Tuberculosis Programme, World Health Organization, Geneva, Switzerland; 7 Health Economics Unit and Division, School of Public Health and Family Medicine, Faculty of Health Sciences, University of Cape Town, Cape Town, South Africa

**Keywords:** tuberculosis services, cost analysis, Kenya

## Abstract

**BACKGROUND:**

The reduction of Kenya’s TB burden requires improving resource allocation both to and within the National TB, Leprosy and Lung Disease Program (NTLD-P). We aimed to estimate the unit costs of TB services for budgeting by NTLD-P, and allocative efficiency analyses for future National Strategic Plan (NSP) costing.

**METHODS:**

We estimated costs of all TB interventions in a sample of 20 public and private health facilities from eight counties. We calculated national-level unit costs from a health provider’s perspective using bottom-up (BU) and top-down (TD) approaches for the financial year 2017–2018 using Microsoft Excel and STATA v16.

**RESULTS:**

The mean unit cost for passive case-finding (PCF) was respectively US$38 and US$60 using the BU and TD approaches. The unit BU and TD costs of a 6-month first-line treatment (FLT) course, including monitoring tests, was respectively US$135 and US$160, while those for adult drug-resistant TB (DR-TB) treatment was respectively US$3,230.28 and US$3,926.52 for the 9-month short regimen. Intervention costs highlighted variations between BU and TD approaches. Overall, TD costs were higher than BU, as these are able to capture more costs due to inefficiency (breaks/downtime/leave).

**CONCLUSION:**

The activity-based TB unit costs form a comprehensive cost database, and the costing process has built-in capacity within the NTLD-P and international TB research networks, which will inform future TB budgeting processes.

## Abstract (fr)

**CONTEXTE:**

Pour réduire le poids sanitaire de la TB au Kenya, il convient d’améliorer l’allocation des ressources au programme national de lutte contre la TB, la lèpre et les maladies respiratoires (NTLD-P), ainsi qu’au sein de celui-ci. Nous avons cherché à estimer les coûts unitaires des services antituberculeux pour que le NTLD-P puisse établir son budget et analyser l’allocation efficace des ressources pour l’estimation future des coûts du Plan stratégique national (NSP).

**MÉTHODES:**

Nous avons estimé les coûts de toutes les interventions de lutte contre la TB auprès d’un échantillon de 20 établissements de santé publics et privés de huit comtés. Nous avons calculé les coûts unitaires au niveau national du point de vue du fournisseur de soins à l’aide des approches ascendante (BU) et descendante (TD) pour l’exercice budgétaire 2017–2018 en utilisant Microsoft^®^ Excel^®^ et STATA v16.

**RÉSULTATS:**

Le coût unitaire moyen de la recherche passive de cas (PCF) était respectivement de 38 $ US et 60 $ US avec les approches BU et TD. Les coûts unitaires BU et TD d’un cycle de traitement de première ligne de 6 mois (FLT), avec tests de contrôle, étaient respectivement de 135 $ US et 160 $ US, alors que ceux du traitement de la TB pharmacorésistante (DR-TB) chez l’adulte étaient respectivement de 3 230,28 $ US et 3 926,52 $ US pour le schéma court de 9 mois. Les coûts des interventions ont mis en évidence des variations entre les approches BU et TD. Dans l’ensemble, les coûts TD étaient plus élevés que les coûts BU, puisque ces derniers peuvent tenir compte de davantage de coûts d’inefficacité (matériel cassé/période d’arrêt/congés).

**CONCLUSION:**

Les coûts unitaires des activités de lutte contre la TB forment une base de données exhaustive des coûts, et le processus de calcul des coûts peut être intégré au NTLD-P et aux réseaux de recherche internationaux sur la TB, ce qui permettra d’orienter les prochains processus d’établissement des coûts de la TB.

TB occurs worldwide and is one of the leading causes of death. In 2018, 84% of new cases occurred in the 30 high TB burden countries (HBCs), including Kenya.^1^ A recent national TB prevalence survey found an incidence of 267 TB cases per 100,000 people, with 40% of TB cases remaining undetected and untreated.^1^ In the past 2 years, global efforts and a declaration have supported countries in accelerating the progress towards the End TB Strategy.^2^

Addressing Kenya’s TB burden requires considerable investment in TB services and interventions, including improving resource allocation both to and within TB programmes.^3^ Global and national funders are required to ensure that TB funding is justified on the best available evidence, such as economic analyses, for sound investments. However, analyses are often uncertain or limited due to cost data scarcity,^4^ for example, on TB services. Cunnama et al. found only three Kenyan studies which reported economic costs of diagnostic and treatment strategies from provider and societal perspectives.^5^ Only seven HBCs have published cost estimates for drug-susceptible TB (DS-TB) treatment from 2010, and cost data on drug-resistant TB (DR-TB) treatment are available for only eight low- and middle-income countries (LMICs).^6^,^7^ Only China, India and South Africa have data for diagnostics beyond smear, culture or chest X-ray (CXR), which vary dramatically by context and placement.^8^

Cost data are essential to inform decisions around efficiency and value for money, and several planning and economic tools currently provide default data, extrapolating from the very limited number of current studies to support countries’ allocation of TB resources.^9^ The WHO-CHOICE (World Health Organization CHOosing Interventions that are Cost Effective) project provides country-specific health service cost estimates based on modelling utilising primary and secondary data. However, these default estimates are may be unreliable due to the limited good-quality country unit cost data available.^10^ A recent National Strategic Planning (NSP) costing survey conducted in Kenya for 2019–2023 had to rely on modelled costs to assess the cost-effectiveness of different interventions.^11^

This study aims to estimate the unit costs of a comprehensive set of TB services that can be used by the NTLD-P for more informed budgeting and planning, and for future NSP costing.

## METHODS

### Study setting

Situated in East Africa, Kenya is administratively divided into 47 counties, with a population of approximately 47.6 million (estimated for 2019).^12^ The prevalence of TB is high, and there were an estimated 158,000 new cases in 2017.^11^

### Sampling

The Value-TB Kenya study was conducted in eight counties from three major regions: Nairobi, Eastern and Western. The counties were purposively sampled to reflect a high, medium and low burden of TB, respectively, based on population size and TB caseload. The study sample size was computed using the approach for unit costs proposed by Johnston et al. Assuming a 95% confidence level and a standard deviation of 25% of the average cost estimate, 24 facilities were estimated as sufficient to give an estimate with a precision of ± 10%.^13^ A standardised, stratified random sampling strategy shared across the countries participating in the Value-TB project was used to select the health facilities.

The sampling frame was created from a national list of healthcare facilities that offer TB services, inclusive of the region (rural and urban), county, facility type, and diagnostic interventions available, and an indicator of the facility size based on TB-specific case workload (Table 1). The inclusion criterion was health facilities that were providing TB treatment in July 2017 (*n* = 3,690 health facilities), as per the list provided by the NTLD-P. We excluded prisons (*n* = 52), because the unit of measure was health facilities.

**Table 1:** Characteristics of sampled facilities (n = 20)

Facility type	Rural facilities *n*	Urban facilities *n*	Public facilities *n*	Private for profit facilities *n*	Faith-based organisation *n*	Total number of sites	Total number TB patients in 2018
Health centre	3	5	5	2	1	8	460
Primary hospital	4	5	7	1	1	9	1800
Secondary hospital	0	2	1	1	0	2	949
Laboratory	0	1	1	0	0	1	NA
Total	7	13	14	4	2	20	3209

NA = not applicable.

### Data collection

Data were collected by four trained researchers between May and November 2018 using publicly available Value TB standard tools (LSHTM Research Online, London, UK ) and checklists in line with the Global Health Cost Consortium’s (GHCC) reference case.^14^ Data were collected retrospectively for the financial year 2017–2018 (July to June) using two primary sources: financial and administrative document reviews from county and facility levels, and interviews with front line workers and managers for time and resource use over the previous month.

### Costing approach

Methods for cost data collection were adapted from Costing Guidelines for Tuberculosis Interventions and Value TB protocol templates^15^ using a health provider’s perspective. Full financial and economic costs were collected retrospectively and reflected ‘real world’ implementation of interventions. The time horizon was one patient episode of care. No start-up costs or costs of supporting change were included (e.g., the costs of pilots and technical assistance). Estimation of future savings, above service level costs, research costs and other unrelated costs were excluded. The broad Value TB costing approach is described further by Sweeney et al.^16^

TB interventions (Supplementary Data 2) and unit costs were identified and defined using the GHCC nomenclature and Costing Guidelines for Tuberculosis Interventions (Supplementary Data 3).^15^ We adopted both top-down (TD) and bottom-up (BU) approaches to allow comparison of unit costs. In BU costing, we identified and valued all the ingredients required to offer services and aggregated the values; while in the TD approach we identified an overall cost incurred and allocated the costs to the various units based on a criteria related to the utilisation of each unit.^15^ Staff costs were estimated using direct observation, short-structured interviews and time-sheets, and valued using county-specific wages. Prices of medical supplies were sourced from local companies. Capital costs were inclusive of maintenance costs and were annuitized at a 3% discount rate (Supplementary Data 4).^17^,^18^

Costs were collected in Kenyan shillings (KES) based on 2017–2018 financial year prices and salaries and are reported in US dollars (USD). Costs obtained outside of this financial year were inflated/deflated using the local consumer price index (CPI) of Kenya (1.05^19^) before converting to USD. An average exchange rate for the year of cost data collection (2018) was used for conversion to USD (US$1 = KES101.29).^20^

### Data analysis

Data completeness was assessed using Stata (Stata, College Station, TX, USA), and data cleaning done in Microsoft Excel (Microsoft, Redmond, WA, USA). Using a dataset that contained all costs for each site, we collapsed the different population groups (pulmonary TB [PTB] vs. extra-pulmonary TB [EPTB]; adults vs. children) and calculated the average unit costs per intervention. The TB services provided are similar for all population groups, except for drug regimen during treatment.

### Ethical approval

The study was approved by Kenya Medical Research Institute (KEMRI) Scientific and Ethics Review Unit, Nairobi (reference: KEMRI/SERU/CGMR-C/111/3603); Council of Governors, Kenya, National Commission for Science, Technology and Innovation, Nairobi, Kenya (NACOSTI; serial no. A17531), and committees at London School of Hygiene & Tropical Medicine, London, UK (reference: 14702) and the University of Cape Town, Cape Town, South Africa (reference: HREC025/2018) and review exemption from WHO AFRO (reference: AFR/ERC/2018/03/01).

## RESULTS

### Average unit cost per intervention

Table 2 gives the average unit cost per intervention. These unit costs highlighted variations between BU and TD approaches such as differences of US$9 for active case-finding (ACF), US$21.95 for passive case-finding (PCF) and US$24.72 for first-line treatment (FLT). The cost of the 9-month, short DR-TB treatment regimen observed in adults was respectively US$3,230.28 and US$3,926.52 with the BU and the TD approaches, and respectively US$9,996.18 and US$8,163.22 for the 18-month long regimen.

**Table 2:** Average unit cost per intervention in 2018 US$

Intervention	Observations^*^ *n*	Bottom-up approach		Top-down approach	
	
		Mean ± SD	95% CI	Mean ± SD	95% CI
BCG vaccination	17	2.46 ± 1.16	1.92–3.02	2.90 ± 1.35	2.25–3.54
ACF^†^	20	40.38 ± 56.52	15.68–65.22	49.38 ± 76.89	15.77–83.16
ICF;^†^ cough triage^‡^	3	1.79 ± 0.47	1.26–2.32	6.48 ± 3.62	2.38–10.57
ICF^†^ finding; screening	70	4.15 ± 3.98	3.23–5.10	4.52 ± 3.11	3.79–5.24
PCF^†^	80	38.32 ± 20.14	33.97–42.80	60.27 ± 36.62	52.35–68.40
First-line treatment^†^	144	134.97 ± 70.94	123.62–146.80	159.69 ± 92.33	144.89–175.05
Second-line short regimen TB treatment (9 months)^†^	3	3,230.28 ± 1,069.15	2,020.45–4,440.12	3,926.52 ± 996.61	2,798.77–5,054.27
Second-line long regimen TB treatment (18 months)^†^	2	9,996.18 ± 6,284.09	1,287.03–18,705.34	8,163.22 ± 2,899.82	4,144.35–12,182.09

^*^ Observations of the different population groups across all sampled facilities for all interventions, except ICF cough triage.

^†^This includes all population groups (adults and children; PTB and EPTB).

^‡^Cough triage observations (*n*) represent the number of facilities (3) observed.

SD = standard deviation; CI = confidence interval; BCG = bacille Calmette-Guérin; ACF = active case-finding; ICF = intensified case-finding; PCF = passive case-finding; PTB = pulmonary TB; EPTB = extrapulmonary TB.

### Unit costs per output for TB interventions

Unit costs per output for TB interventions are given in Supplementary Data 6. BCG vaccinations were predominantly delivered at the facility level, with only three facilities conducting community outreaches.

ACF mainly comprised contact tracing through one home visit costing respectively US$13.54 and US$15.52 using the BU and the TD approach. Community outreach events conducted by six facilities utilising mobile clinics, consisted of TB screening and sputum sample collection, at a unit BU and TD cost of respectively US$108.65 and US$125.44. Generally, one nurse, a community health volunteer (CHV), a laboratory technician and driver were involved in these mass TB screenings.

In intensive case-finding (ICF), 100% of newly enrolled and in-care HIV-positive individuals and high-risk population (mothers in antenatal care [ANC]) were screened for TB. CHVs observed and triaged ‘coughers’ in the clinic waiting areas. Both populations were screened in outpatient visits using the WHO-recommended TB symptoms screening tool. For PCF, the cost per TB case diagnosed consisted of outpatient screening and diagnostic visits. TB diagnostic tests and other laboratory tests were performed per TB case diagnosed. CXR and Xpert^®^ MTB/RIF (Cepheid, Sunnyvale, CA, USA) tests were less available in rural facilities. Costs were excluded when Xpert samples were transported to other laboratories or when patients were sent to other facilities for CXR. For inpatient visits, we estimated the BU and TD bed-days across both diagnosis and treatment cases, at respectively US$20.11 and US$23.28.

The common outputs for DS-TB and DR-TB treatment were outpatient treatment visit, inpatient bed-days, lost to follow-up (LTFU) tracing (involving home visits and/or phone calls by CHVs) and CXR. Nutritional support consisting of an average of two visits per patient,^21^ and food supplements was the largest contributor costing respectively US$42.58 and US$50.67 as per the BU and TD approach. Data on the DS-TB treatment visits were per protocol, which is the current practice.^*^ DR-TB treatment also included drug-resistant tests and additional follow-up tests where TB sputum samples were processed in the National TB Reference Laboratory (NTRL); other laboratory tests were processed at Lancet Laboratories, Nairobi, Kenya. Therefore, follow-up tests were not facility-incurred costs and were not captured in the cumulative unit cost of DR-TB treatment.

TB prevention treatment (TPT) outputs comprised an outpatient screening visit, diagnostic visit and outpatient TPT visits costing respectively US$3.84 and US$4.13 as per the BU and the TD approach. The procurement of drugs for TB treatment and prevention was centralised at the programme level; costs were incurred by the NTLD-P which provided the medications to facilities. The drug unit costs were included in the intervention costs, and were US$23 for adult PTB and EPTB, US$31 for child PTB and EPTB on DS-TB regimens, US$1,188 for the short DR-TB regimen, US$3,336 for the long DR-TB regimen, US$6 for TPT for HIV-positive adults and US$4 for children contacts aged <5 years.^†^

### Proportion of cost categories contributing to the outputs per TB intervention

**Figure 1.**
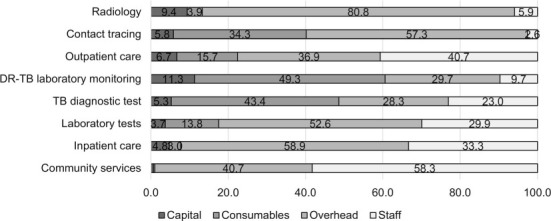
Proportion of cost categories contributing to the outputs of TB interventions. DR-TB = drug-resistant TB.

**Figure 2.**
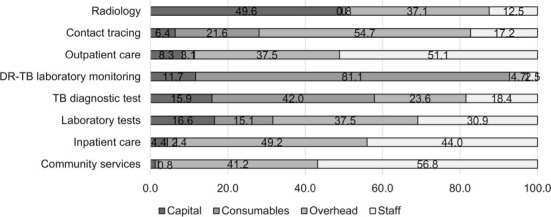
Proportion of inputs contributing to the outputs of TB interventions. DR-TB = drug-resistant TB.

The proportion of cost categories contributing to the outputs varied between both costing approaches (Figures 1 and 2). Staff costs were the main cost drivers of all outpatient and community service outputs, and inpatient service outputs for the TD approach. These outputs are labour-intensive, and most patients received facility-based treatment while community visits were reported for very few patients (three patients) and included well reported travel and home visit time estimates.

Capital costs comprised a higher proportion of costs for TD than BU estimates, specifically observed for radiology. Consumable costs for DR-TB laboratory tests showed significantly higher TD costs than BU, which result from lower number of samples processed, as indicated during an upgrade of the information digital system in this period. Overall, the TD cost estimates were higher than BU estimates, which is to be expected as they capture more costs due to inefficiency (such as breaks, downtime and leave) than BU.

## DISCUSSION

The Value-TB study represents one of the most thorough endeavours to comprehensively cost all TB interventions across different population groups. Our study is among the growing number of costing studies for TB services, but is unique in providing country-specific cost estimates based on primary data that reflect current practices. This is important to inform decisions on efficiency and value for money for investments at the programme level.

As expected, TD unit cost estimates were consistently higher than BU estimates. This could be attributed to staff capacity who may be involved in other clinic tasks; it is also possible that the TD estimate captures staff downtime for PCF and DR-TB treatment. In comparison, 100% of the HIV-positive population and high-risk groups were screened for TB,^22^ resulting in very little staff downtime and a reduced difference between TD and BU (US$0.35) cost estimates.

FLT costs were substantially higher in similar settings such as South Africa (US$256.61^*^)^23^ and Nigeria (US$227.14^†^)^24^ compared to our study findings (BU: US$135; TD: US$160). These studies included the additional costs for adverse drug reactions (drugs, extra monitoring tests and clinic visits), which may explain the variation. The long DR-TB treatment regimen cost (BU: US$9,996.18; TD: US$8,163.22) was lower in our study compared to Russia (US$14,600^‡^),^25^ but higher than South Africa (US$6,712^*^),^23^ Peru (US$2,400^§^) and the Philippines (US$3,613^¶^).^25^ These differences reflect dissimilarities in drug prices, laboratory test prices, hospitalisation costs, and also exclusion and inclusion of cost components.^25^ The nationally representative granular empirical costing that we have undertaken, as well as the breakdown of costing components allows for the generation of a range of costs, which are likely to be truer estimates of the unit costs of TB services.

We found that nutritional support was a cost driver for DS-TB treatment (36%) unit cost in Kenya. However, in 2019, 46% of DS-TB patients were found to be malnourished and 56% of adult DR-TB patients were undernourished.^26^ With poor nutrition as one of the drivers of the TB epidemic in Kenya,^26^,^27^ adequate investment and continued efforts to improve nutrition among TB patients is important.

Our study reports the available TB diagnostic tests and the associated factors for TB diagnostics. Health facilities in urban areas offered a wider combination of diagnostic tests, and these costs were higher compared to rural facilities. This resulted in the referral of patients to urban facilities for diagnostic and additional tests not available at rural facilities at the patient’s expense. The fact that only a few facilities had Xpert diagnostics may have resulted in patient delays. The NTLD-P annual report states that only 47% of notified TB cases have access to Xpert testing.^26^ In addition, finding missing TB cases is an important strategy in bridging the TB case detection gap.^11^

We also found that only eight community-based activities for finding TB cases occurred in the sampled regions over a 1-year period, which would explain the relatively high cost per community event, particularly where diagnostic test capacity such as CXR was under-utilised. However, when scaled-up, community-based activities like outreach visits to create awareness, mass screening in high-risk communities and the use of mobile screening units, have been found to be cost-saving,^28^–^30^ with potential for substantial efficiency gains by the NTLD-P.

Our study had several limitations. Apportioning capital items specific to TB services for the laboratory and radiology departments posed a methodological challenge because the equipment is utilised for all patients. Estimation of the cost of inpatient visits was limited by the documentation format of inpatient records, and we estimated bed-days across both diagnosis and treatment cases. In some cases, we were limited to the services available in the sampled facilities; therefore, we did not cost diagnosis, treatment and care for extensively drug-resistant TB (XDR-TB). A strength of this analysis is the comprehensive cost dataset collected based on both BU and TD approaches to improve accuracy of the estimations. TD costs may be utilised for budgeting purposes and BU costs in modelling the most efficient practice.

There remain several important research opportunities. Limited availability of cost data on ‘new’ approaches will be critical to the achievement of NTLD-P goals.^31^,^32^ Our findings take into account all activities contributing to an intervention, including non-financial data such as time spent providing TB services by healthcare workers (including volunteers), which captures efficiency levels across both costing approaches (BU and TD). This improves the quality of cost data available for planning and budgeting.

The policy recommendations from the facility-level costs of TB services are outlined below. First, this data will benefit the NSP costing process for all TB interventions to ensure adequate and efficient investments are made in areas of TB diagnosis and treatment. Second, community-based events such as mass screening in high-risk communities that have proven to be low-cost activities, and which may be scaled up, greatly impact identification of missing TB cases and improve the case detection rate in line with the End-TB Strategy.^33^ Third, the activity-based costs for our study may be beneficial in the subsequent NSP development and donor funding applications, as it considers efficiency and value for money aspects. In conclusion, these unit costs form a comprehensive cost database that may inform future TB budgeting processes in Kenya and contribute to existing literature on TB cost data.

## Footnotes

^*^ 1USD = South African rand 7.05, 2011.

^†^ 1USD =Nigerian naira 118.5, 2008.

^‡^ 1USD = Russian rouble 28.67, 2005.

^§^ 1USD = Peruvian sol 3.25, 2005.

^¶^ 1USD = Philippine peso 55.85, 2005.
